# Strengthening laboratory systems for ensuring accurate diagnoses in mother-to-child transmission (MTCT) prevention programs in Uganda: a narrative review

**DOI:** 10.1097/MS9.0000000000002154

**Published:** 2024-05-08

**Authors:** Emmanuel Ifeanyi Obeagu, Getrude Uzoma Obeagu

**Affiliations:** a Department of Medical Laboratory Science; b School of Nursing Science, Kampala International University, Kampala, Uganda

**Keywords:** antenatal care, HIV diagnosis, laboratory systems, maternal health, MTCT prevention, Uganda, vertical transmission

## Abstract

Mother-to-child transmission (MTCT) of HIV remains a significant public health challenge in Uganda, necessitating a focused examination of the state of laboratory systems to ensure accurate diagnoses and effective prevention. The aim of this narrative review is to assess the current state of laboratory systems supporting MTCT prevention programs in Uganda, identify challenges hindering accurate diagnoses, and propose strategies for strengthening these systems to enhance the effectiveness of MTCT prevention efforts. This narrative review explores the current landscape of laboratory infrastructure in Uganda, addressing challenges unique to the country and proposing strategies for improvement. The discussion encompasses the integration of molecular testing, the role of point-of-care diagnostics, the implementation of quality assurance programs, and capacity-building initiatives for laboratory personnel. Additionally, technological innovations and their applicability in the Ugandan context are explored alongside the crucial aspect of integrating laboratory services into antenatal care. Drawing on global lessons, the review provides tailored recommendations for Uganda, spanning policy considerations, funding mechanisms, infrastructure enhancements, and workforce development. Looking towards the future, the review outlines potential collaborations, technological advancements, and strategic investments that can further fortify laboratory systems, ultimately contributing to the elimination of MTCT in Uganda.

## Introduction

Highlights

- Importance of reliable diagnoses: Accurate diagnosis is crucial for effective mother-to-child transmission (MTCT) prevention programs.
- Challenges in laboratory infrastructure: Uganda faces challenges in laboratory infrastructure, including inadequate equipment, limited access to quality reagents, and a shortage of skilled personnel.
- Role of point-of-care testing (POCT): Point-of-care testing can overcome some of the challenges associated with traditional laboratory testing by providing rapid results.
- Need for integration and collaboration: Strengthening laboratory systems for MTCT prevention requires collaboration between stakeholders at various levels.

Mother-to-child transmission (MTCT) of HIV remains a critical public health concern, particularly in regions with high prevalence rates such as Uganda. Despite significant progress in global efforts to combat the HIV epidemic, MTCT persists as a major obstacle to achieving an AIDS-free generation.^1,2^ The success of prevention programs relies heavily on accurate and timely diagnoses, underscoring the pivotal role of laboratory systems in this endeavor. This paper sets the stage for a narrative review that aims to examine the current state of laboratory systems in Uganda, identifying challenges specific to the context and proposing strategies for strengthening these systems to ensure accurate diagnoses for effective MTCT prevention. Uganda has been significantly affected by the HIV epidemic, with a prevalence rate that has posed substantial challenges to public health initiatives. While progress has been made, particularly in reducing overall HIV prevalence, the persistence of MTCT demands focused attention. Understanding the unique dynamics of the HIV epidemic in Uganda is crucial for tailoring interventions and strengthening healthcare systems.^3^

Accurate and timely diagnosis is fundamental to the success of MTCT prevention efforts. Laboratory systems play a central role in detecting and monitoring HIV in pregnant women, facilitating timely interventions to prevent vertical transmission.^4^ The paper underscores the critical need to assess and enhance laboratory capabilities in Uganda to effectively combat MTCT. This review aims to provide a comprehensive analysis of the current state of laboratory systems in Uganda concerning MTCT prevention. By identifying challenges specific to the country, exploring advancements in diagnostics, and proposing strategies for improvement, the review seeks to contribute to the broader discourse on global efforts to eliminate MTCT.

The medical system in Uganda faces various challenges but has also seen significant improvements over the years. Uganda’s healthcare infrastructure is a mix of public and private facilities. The public sector includes government-run hospitals, health centers, and dispensaries, which serve the majority of the population, especially in rural areas. Private facilities, including hospitals and clinics, cater to those who can afford higher-quality care. Uganda faces a shortage of healthcare professionals, including doctors, nurses, and specialists. This shortage is particularly acute in rural areas, leading to uneven distribution of healthcare workers and limited access to medical services for many communities. Uganda grapples with a high burden of communicable diseases such as HIV/AIDS, malaria, tuberculosis, and neglected tropical diseases. Non-communicable diseases (NCDs) are also on the rise, presenting new challenges for the healthcare system. The country has made strides in combating diseases like HIV/AIDS, with the implementation of prevention, treatment, and care programs. However, challenges persist, including ensuring access to antiretroviral therapy (ART), reducing mother-to-child transmission of HIV, and addressing stigma and discrimination. Maternal and child health indicators have improved in recent years, but maternal mortality rates remain high. Access to prenatal care, skilled birth attendance, and postnatal services are crucial areas for improvement. Uganda’s healthcare system is predominantly financed through public funding, supplemented by external aid and out-of-pocket payments. However, funding constraints often limit the government’s ability to provide adequate resources for healthcare delivery and infrastructure development. Data collection, management, and reporting systems in Uganda’s healthcare sector are improving but still face challenges related to accuracy, timeliness, and interoperability between different health information platforms. Traditional medicine plays a significant role in Uganda’s healthcare landscape, especially in rural areas, where it is often more accessible and affordable than modern healthcare services. Efforts to integrate traditional medicine into the formal healthcare system are ongoing.^4^

This review holds significance in guiding policymakers, healthcare providers, and researchers in Uganda by offering insights into the specific needs and opportunities for strengthening laboratory systems. It is anticipated that the findings and recommendations presented will inform evidence-based strategies for optimizing diagnostic accuracy, ultimately contributing to the broader goal of eliminating MTCT in Uganda.

## Aim

The aim of this narrative review is to assess the current state of laboratory systems supporting MTCT prevention programs in Uganda, identify challenges hindering accurate diagnoses, and propose strategies for strengthening these systems to enhance the effectiveness of MTCT prevention efforts.

## Methodology

Different research search databases, such as Pubmed central, Scopus, Web of Science, Medline, Google Scholar etc, were utilized in writing this paper. The review is structured to cover various aspects critical to laboratory system strengthening. This includes an examination of existing challenges, advancements in molecular testing, the role of point-of-care diagnostics, quality assurance programs, capacity-building initiatives, technological innovations, and the integration of laboratory services into antenatal care. Drawing on global experiences, the review concludes with tailored recommendations for Uganda, providing a roadmap for future interventions.

### Current landscape of laboratory systems in Uganda

The laboratory infrastructure in Uganda, vital for supporting comprehensive healthcare services, plays a pivotal role in addressing the challenges posed by MTCT of HIV. Uganda’s laboratory infrastructure exhibits a varied landscape.^5^ While urban areas may have relatively well-equipped laboratories, rural regions face significant challenges, including limited access to diagnostic facilities. This urban-rural disparity underscores the importance of targeted interventions to ensure equitable access to laboratory services. A critical component of laboratory systems is the human capital.^6^ In Uganda, shortages in trained laboratory personnel, especially in remote areas, pose challenges to maintaining consistent and high-quality diagnostic services. Strengthening workforce capacity through training programs and incentives is essential for overcoming this hurdle. The availability and functionality of laboratory equipment vary across different regions of Uganda.^7^ While some laboratories are equipped with advanced technologies for HIV testing, others may rely on more conventional methods. A comprehensive review of existing equipment, ensuring maintenance, and strategic investments in modern technologies are imperative to standardize diagnostic capabilities.

The integration of laboratory systems into broader healthcare structures is crucial for the effectiveness of MTCT prevention programs.^8^ In Uganda, achieving seamless coordination between laboratories and antenatal care services remains an ongoing challenge. Strengthening linkages and optimizing data-sharing mechanisms can enhance the overall efficiency of the healthcare delivery system. Effective data management is vital for tracking the progress of MTCT prevention efforts.^9^ Challenges in data collection, reporting, and analysis persist, affecting the accuracy of program monitoring. Implementing robust information systems, coupled with training for data handlers, is essential for improving the reliability of data related to MTCT. Quality assurance programs are instrumental in ensuring the accuracy and reliability of laboratory results.^10^ In Uganda, the implementation of standardized protocols and accreditation mechanisms is essential for upholding the quality of diagnostic services. Continuous monitoring and evaluation are critical components of a quality assurance framework. Remote and conflict-affected areas in Uganda face unique challenges in establishing and maintaining laboratory services.^11^ Issues such as limited infrastructure, security concerns, and restricted accessibility hinder the delivery of timely and accurate diagnostic services. Tailored strategies and humanitarian interventions are essential to address these specific challenges. Engaging the private sector can contribute significantly to strengthening laboratory systems in Uganda.^12^ Collaborative efforts between public and private entities, along with transparent regulatory frameworks, can enhance resources, infrastructure, and service delivery.

### Challenges specific to Uganda

While Uganda has made significant strides in addressing the HIV epidemic, several challenges unique to the country’s context pose obstacles to the effective functioning of laboratory systems for MTCT prevention. Resource constraints, both financial and infrastructural, are pervasive challenges in Uganda.^13^ Many laboratories, particularly those in rural and underserved areas, face limitations in funding, equipment, and human resources. Adequate investment is crucial to bridge these gaps and build a resilient laboratory infrastructure. Uganda’s diverse geography presents challenges in ensuring uniform access to laboratory services.^14^ Remote and rural areas often lack well-equipped laboratories, leading to disparities in diagnostic capabilities. Addressing geographical discrepancies requires targeted interventions to extend laboratory services to underserved regions.

A shortage of trained laboratory personnel is a significant challenge.^15^ Laboratories, especially in remote areas, struggle to maintain an adequate workforce, impacting the quality and consistency of diagnostic services. Investing in education and training programs for laboratory professionals is essential to overcome this human resource deficit. The transportation infrastructure in Uganda, particularly in remote areas, poses challenges for the timely and secure transfer of samples to laboratories.^16^ Delays in sample transportation can lead to compromised sample integrity and hinder the accuracy of diagnostic results. Improving logistics and investing in transportation networks are critical for overcoming this challenge. Sociocultural factors, including stigma and discrimination, continue to influence HIV-related health-seeking behavior in Uganda.^17,18^ This can deter pregnant women from accessing antenatal care and undergoing HIV testing. Addressing these factors through community engagement and awareness campaigns is essential to increase testing rates and improve MTCT prevention outcomes.

The integration of laboratory services with broader healthcare systems, particularly antenatal care, faces challenges in Uganda.^19^ Siloed approaches and insufficient coordination can lead to missed opportunities for early diagnosis and intervention. Enhancing the integration of services is vital for optimizing MTCT prevention efforts. Historical political and economic challenges have at times affected the stability and functionality of healthcare systems in Uganda.^20^ Periods of instability can disrupt the supply chain, hinder infrastructure development, and impact the overall effectiveness of laboratory systems. Ensuring political and economic stability is crucial for sustained progress in MTCT prevention. Public awareness about the importance of early HIV testing and MTCT prevention measures is often insufficient.^21^ This lack of awareness contributes to late presentations for antenatal care and testing, reducing the effectiveness of preventive interventions. Implementing targeted public awareness campaigns can help address this challenge.

The lack of robust data management systems hampers effective monitoring and evaluation of MTCT prevention programs.^22^ Incomplete or inaccurate data can impede evidence-based decision-making and hinder the assessment of program impact. Strengthening data management systems is critical for improving program outcomes.

### Advances in molecular testing

Molecular testing has emerged as a cornerstone in the prevention of MTCT of HIV, offering heightened sensitivity, specificity, and efficiency in diagnosing infections during pregnancy. In the Ugandan context, where the fight against MTCT is ongoing, embracing and implementing these advances in molecular testing is critical. Polymerase Chain Reaction (PCR) technology remains pivotal in Uganda’s MTCT prevention programs, enabling early infant diagnosis.^23^ Advances in PCR techniques allow for the detection of HIV DNA or RNA in newborns, enabling rapid initiation of ART if necessary. Implementing widespread access to EID PCR testing is crucial for early identification and intervention. Recent advancements include the development of point-of-care nucleic acid testing (NAT), offering the potential for rapid and on-site HIV testing.^24^ These technologies, characterized by their portability and quick turnaround times, can significantly enhance accessibility to testing, particularly in remote or resource-limited settings in Uganda.

Next-generation sequencing (NGS) technologies present an opportunity to revolutionize HIV diagnostics. In Uganda, the application of NGS can provide detailed insights into viral diversity, helping tailor treatment strategies. NGS can contribute to the understanding of HIV transmission dynamics and guide interventions for MTCT prevention.^25,26^ Accurate monitoring of viral load is crucial for ensuring the effectiveness of ART. Molecular testing methods, such as quantitative PCR, offer a precise measurement of viral RNA levels. Scaling up access to routine viral load monitoring supports timely adjustments to treatment regimens, reducing the risk of MTCT.^27^ The emergence of drug-resistant strains poses a challenge to effective MTCT prevention. Molecular testing for drug resistance mutations enables the identification of resistance patterns, guiding clinicians in selecting the most efficacious antiretroviral drugs for pregnant women in Uganda.^28^

While molecular testing advancements offer promising outcomes, their successful integration into Ugandan healthcare systems faces challenges.^29^ These include the need for specialized training, infrastructure investments, and considerations for the decentralization of testing services to reach remote populations. The costs associated with molecular testing technologies can be a barrier to widespread adoption, particularly in resource-limited settings.^30^ Balancing the benefits against financial constraints is a critical consideration for sustainable implementation in Uganda. The successful integration of molecular testing relies on a skilled workforce. Capacity-building initiatives and ongoing training programs for laboratory personnel in Uganda are essential to ensure accurate test performance, result interpretation, and effective utilization of these technologies.^31^ Maintaining high standards of quality assurance is paramount for the reliability of molecular testing.^32^ Establishing and reinforcing quality control measures, proficiency testing, and external quality assessment programs are vital for ensuring the accuracy and precision of test results in Uganda. Community awareness and understanding of molecular testing’s benefits are crucial for its acceptance and success. Implementing community engagement and education programs can enhance awareness, reduce stigma, and encourage the uptake of testing services in Uganda.

### Point-of-care diagnostics in Uganda

Point-of-care (POC) diagnostics have emerged as a transformative approach in healthcare, particularly in resource-limited settings like Uganda. In the context of MTCT prevention, POC diagnostics play a crucial role in enhancing accessibility, reducing turnaround times, and improving overall efficiency. Implementing POC diagnostics for rapid HIV testing at antenatal clinics is a cornerstone of Uganda’s MTCT prevention strategy.^33^ Rapid tests, such as lateral flow assays, enable same-day results, facilitating prompt initiation of ART for HIV-positive pregnant women. This approach increases the likelihood of successful prevention of vertical transmission. Extending POC diagnostics to early infant diagnosis is critical for the timely identification of HIV in newborns. Techniques like dried blood spot testing or nucleic acid testing at the point of care allow healthcare providers in Uganda to rapidly determine an infant’s HIV status, enabling swift intervention and treatment.^33^

POC diagnostics for CD4 count and viral load monitoring offer real-time insights into the immune status and viral suppression of pregnant women living with HIV. These technologies empower healthcare providers in Uganda to make informed decisions regarding ART adjustments, ensuring optimal maternal health and reducing the risk of MTCT.^34^ The seamless integration of POC diagnostics with antenatal care services is paramount for success. Ensuring that diagnostic tools are readily available within antenatal clinics facilitates immediate testing and intervention, overcoming potential delays associated with centralized laboratory testing.^34^ While POC diagnostics hold great promise, challenges persist in extending these technologies to remote and underserved areas of Uganda. Limited infrastructure, unreliable power supply, and transportation constraints can hinder the effective implementation of POC diagnostics in these regions. Maintaining the accuracy and reliability of POC diagnostics requires robust quality assurance programs. Ongoing training for healthcare workers ensures proper usage, result interpretation, and adherence to testing protocols, contributing to the overall effectiveness of POC diagnostic implementation in Uganda.^33^ Balancing the benefits of POC diagnostics against cost implications is crucial for sustainability. While POC tests may reduce the overall time and cost associated with diagnosis and intervention, their initial setup and maintenance costs should be carefully evaluated in the Ugandan context. Community engagement and building trust are integral components of successful POC diagnostic programs. Transparent communication, addressing community concerns, and involving local stakeholders in the implementation process contribute to the acceptance and uptake of POC diagnostics in Uganda.^33^ Effective POC diagnostic programs require robust data management systems. Ensuring connectivity and integration with larger healthcare information systems in Uganda is essential for accurate record-keeping, reporting, and monitoring of MTCT prevention outcomes.^34^

### Quality assurance programs

Quality assurance is a critical component of laboratory systems aiming to prevent MTCT of HIV in Uganda. Ensuring the accuracy, reliability, and consistency of diagnostic results is paramount for the success of MTCT prevention programs. Establishing and adhering to standardized testing protocols is fundamental for maintaining consistency in laboratory practices. In Uganda, ensuring that all laboratories involved in MTCT prevention follow standardized protocols for HIV testing, from sample collection to result reporting, is a cornerstone of quality assurance.^35^ Proficiency testing involves regularly assessing the performance of laboratory personnel through external evaluations.[^36^] In Uganda, implementing proficiency testing programs ensures that laboratory staff maintain the necessary skills and competencies for accurate and reliable HIV testing, contributing to ongoing quality assurance efforts. External quality control programs involve the periodic evaluation of laboratory performance by external entities. In Uganda, collaborating with external quality control providers helps verify the accuracy of testing procedures, identify areas for improvement, and maintain the overall quality of MTCT prevention diagnostics.^36^

Ensuring the accuracy of laboratory equipment is crucial for reliable results. Regular calibration and maintenance of testing equipment in Uganda’s laboratories contribute to quality assurance by preventing inaccuracies that may arise from equipment malfunctions or drift over time.^37^ Ongoing training programs for laboratory personnel are essential for maintaining and improving their skills. Capacity-building initiatives in Uganda should focus on continuous education, staying updated on the latest testing methodologies, and reinforcing the importance of adherence to quality assurance protocols.^35^ Conducting internal quality audits within laboratories allows for a systematic review of processes and practices. This self-assessment in Uganda ensures that laboratories proactively identify and rectify any issues that may compromise the quality of HIV testing and MTCT prevention efforts.^36^ Participation in external quality assessment programs involves laboratories in Uganda submitting samples for testing by external entities. Regular participation allows for an external evaluation of laboratory performance, providing valuable feedback and contributing to ongoing quality improvement.^37^ Effective data management is integral to quality assurance. Implementing robust data management systems in Uganda ensures accurate record-keeping, facilitates real-time monitoring of testing outcomes, and supports evidence-based decision-making for MTCT prevention.^38^ Establishing channels for continuous communication and feedback is crucial for quality assurance programs. Regular communication between laboratories, healthcare providers, and quality control entities in Uganda facilitates the exchange of insights, best practices, and corrective actions. Engaging the community in quality assurance initiatives is essential for building trust and transparency. Community awareness programs in Uganda should highlight the importance of quality assurance in ensuring accurate diagnoses and fostering confidence in MTCT prevention efforts.

### Capacity-building initiatives in Uganda

Capacity building is a crucial aspect of strengthening laboratory systems for effective MTCT prevention in Uganda. Enhancing the skills, knowledge, and competencies of laboratory personnel is essential for ensuring accurate diagnostics and improving overall healthcare outcomes. Implementing structured training programs for laboratory personnel in Uganda is essential to equip them with the necessary skills for accurate HIV testing.^39^ These programs should cover a range of topics, including the latest diagnostic techniques, quality assurance protocols, and ethical considerations in MTCT prevention. As new technologies emerge, ensuring that laboratory personnel are adept at using these technologies is crucial. Capacity-building initiatives in Uganda should focus on skill development for handling advanced diagnostic tools, molecular testing methods, and point-of-care technologies relevant to MTCT prevention. Promoting continuous professional development is vital for keeping laboratory personnel updated with the latest advancements in HIV diagnostics and MTCT prevention strategies. Workshops, conferences, and online courses can provide ongoing opportunities for learning and skill refinement.^40^

Establishing mentorship programs pairs experienced laboratory professionals with newer staff, facilitating knowledge transfer and skill development. In Uganda, mentorship initiatives can help bridge the experience gap, ensuring a continuous cycle of learning and expertise enhancement. Collaborating with academic institutions in Uganda fosters a supportive environment for capacity building.^41^ Partnerships can include developing specialized courses, offering internships, and facilitating research opportunities, contributing to a well-rounded education for laboratory personnel. Establishing regional training centers in Uganda ensures that capacity-building initiatives are accessible to personnel across diverse geographical locations. These centers can offer hands-on training, workshops, and collaborative activities to enhance skills in MTCT prevention. Given the interdisciplinary nature of MTCT prevention, providing cross-disciplinary training is essential. Laboratory personnel in Uganda should receive training that spans virology, immunology, obstetrics, and public health to foster a holistic understanding of MTCT dynamics.^38^

Incentive programs can motivate laboratory personnel in Uganda to actively participate in capacity-building initiatives. Recognizing and rewarding achievements, such as certifications or successful implementation of new techniques, can boost morale and encourage ongoing learning. Utilizing accessible e-learning platforms in Uganda facilitates flexible learning opportunities for laboratory personnel. Online courses, webinars, and virtual training sessions offer a convenient way to enhance skills, especially in remote or resource-limited areas.^40^ Engaging laboratory personnel in quality improvement projects empowers them to apply theoretical knowledge to real-world scenarios. In Uganda, encouraging participation in projects related to MTCT prevention fosters a culture of continuous improvement and innovation. Conducting regular workshops and knowledge transfer sessions provides a forum for sharing best practices, addressing challenges, and fostering collaboration among laboratory personnel in Uganda. These sessions can focus on specific aspects of MTCT prevention and diagnostics.^41^ Investing in leadership development programs in Uganda ensures that laboratory personnel can take on leadership roles within their teams. Leadership skills are essential for driving initiatives, implementing changes, and fostering a culture of continuous improvement. Table 1 shows quality assurance measures, and Table 2 shows integration of laboratory services (provided by the authors for this review article)

**Table 1:** Quality assurance measures.

Quality control activity	Frequency	Responsible personnel	Documentation	Follow-up actions
Daily internal quality control	Daily	Medical Laboratory Scientists	QC logbook entries	Immediate corrective action for out-of-range results
External quality assessment	Quarterly	Laboratory Manager	EQA report submission	Review of results, Implementation of corrective measures
Equipment maintenance checks	Monthly	Biomedical Engineers	Equipment maintenance logs	Scheduled maintenance, Calibration checks

**Table 2:** Integration of laboratory services.

Integrated service	Components	Key activities	Benefits
Antenatal care (ANC)	HIV testing, PMTCT counseling	Routine HIV screening during ANC visits, Immediate initiation of PMTCT interventions	Early identification of HIV-positive pregnant women, Reduction in MTCT rates
HIV treatment services	CD4 count monitoring, viral load testing	Regular monitoring of HIV-infected individuals on ART, Early detection of treatment failure	Timely adjustments to treatment regimens, Improved patient outcomes
Maternal and child health clinics	Early infant diagnosis (EID)	Prompt testing of HIV-exposed infants, Immediate initiation of treatment if positive	Reduction in infant morbidity and mortality related to HIV

ART, antiretroviral therapy; MTCT, mother-to-child transmission; PMTCT, Prevention of Mother to Child Transmission.

### Technological innovations in the Ugandan context

Technological innovations have the potential to revolutionize the landscape of MTCT prevention in Uganda.^42^ Embracing cutting-edge technologies can enhance diagnostic accuracy, improve data management, and streamline healthcare delivery. Integrating artificial intelligence into diagnostic processes offers a promising avenue for improving accuracy and efficiency. In Uganda, artificial intelligence (AI) applications can assist in interpreting test results, identifying patterns, and predicting HIV transmission risks, providing valuable insights for healthcare providers and enhancing early intervention strategies.^43^ Leveraging mobile health solutions in Uganda facilitates real-time data collection, patient monitoring, and communication. Mobile applications can be utilized for appointment reminders, medication adherence, and remote consultation, improving overall engagement and retention in MTCT prevention programs.^44^ Telemedicine platforms enable remote consultations between healthcare providers and patients. In Uganda, telemedicine can bridge the gap between urban centers and remote areas, ensuring that pregnant women receive timely advice, counseling, and support for MTCT prevention.^45^ Implementing blockchain technology enhances the security and integrity of health data. In Uganda, where data privacy is paramount, blockchain can ensure secure storage, sharing, and access to patient information, contributing to the establishment of a robust and trustworthy health information system.^42^

Overcoming geographical challenges in Uganda, drones can be employed for the swift and secure transportation of samples from remote areas to laboratories. This ensures timely testing and intervention, particularly for antenatal care services, contributing to the effectiveness of MTCT prevention.^43^ Internet of Things (IoT) devices can monitor the functionality and maintenance needs of laboratory equipment. In Uganda, deploying IoT sensors ensures that diagnostic tools operate optimally, reducing downtime and contributing to the reliability of HIV testing services.^45^ ^n^ Machine learning algorithms can analyze vast datasets to predict trends, risks, and outcomes related to MTCT. In Uganda, these predictive analytics can aid in identifying high-risk populations, optimizing resource allocation, and tailoring interventions for maximum impact.^42^ Augmented reality (AR) applications can enhance training programs for laboratory personnel in Uganda. Interactive and immersive training experiences using AR technology improve skills acquisition, particularly in the context of hands-on tasks and complex procedures related to MTCT prevention.^42^ Biometric identification, such as fingerprint or iris scanning, ensures accurate patient tracking and linkage to care. In Uganda, biometric solutions can enhance the identification of pregnant women living with HIV, facilitating targeted interventions and preventing loss to follow-up.^46^ Wearable devices and remote monitoring technologies offer real-time tracking of maternal health parameters. In Uganda, these innovations can empower pregnant women to actively participate in their healthcare, enabling healthcare providers to intervene promptly in case of any complications.

### Integration with antenatal care services in Uganda

Ensuring the seamless integration of laboratory services with antenatal care (ANC) is pivotal for the success of MTCT prevention programs in Uganda. Integrating early and routine HIV testing into ANC services is a fundamental step.^47^ Ensuring that all pregnant women are offered HIV testing at their first ANC visit and during subsequent visits in Uganda facilitates early identification of HIV-positive mothers, enabling timely interventions to prevent MTCT. Incorporating comprehensive counseling and education services within ANC settings is crucial.^48^ Ugandan healthcare providers should offer counseling on HIV prevention, treatment options, and the importance of adherence, empowering pregnant women to make informed decisions regarding MTCT prevention. Deploying point-of-care testing at ANC clinics enhances accessibility and expedites the diagnostic process. Integrating rapid HIV tests and other relevant point-of-care diagnostics in Uganda allows for immediate results, facilitating prompt initiation of ART and reducing the risk of MTCT.^38^

Establishing strong linkages between ANC services and ART initiation is critical. In Uganda, a seamless referral system ensures that HIV-positive pregnant women identified during ANC are promptly connected to ART services, contributing to the effective prevention of vertical transmission.^47^ Integrating laboratory data with ANC records and overall healthcare information systems ensures holistic patient management. This integration in Uganda enables healthcare providers to track and manage HIV-positive pregnant women throughout the continuum of care, optimizing MTCT prevention efforts.^47^ Promoting family-centered approaches within ANC services in Uganda involves engaging partners and family members in the MTCT prevention process. Encouraging joint HIV testing, involving male partners, and providing family-focused counseling contribute to a supportive environment for pregnant women.^48^ Expanding the scope of ANC laboratory services to include screening for opportunistic infections is beneficial. In Uganda, incorporating tests for conditions such as syphilis and tuberculosis during ANC allows for comprehensive care, reducing the overall health risks for both mothers and infants. Integrating routine viral load monitoring into ANC services is essential for optimizing the management of HIV-positive pregnant women on ART. In Uganda, incorporating viral load testing during ANC visits enables timely adjustments to treatment regimens, ensuring viral suppression and reducing the risk of MTCT.^47^ Leveraging innovative technologies, such as mobile health apps or SMS-based reminders, can enhance ANC support. In Uganda, these technologies can be employed to provide appointment reminders, medication adherence support, and educational resources, improving overall engagement in MTCT prevention.^46^ Implementing task-shifting strategies and training healthcare workers in ANC clinics enhances their capacity to deliver comprehensive MTCT prevention services. In Uganda, providing ANC providers with the necessary skills for HIV testing, counseling, and data management contributes to a well-rounded healthcare workforce. Fostering community involvement and support groups within ANC services encourages peer support and shared experiences. In Uganda, community-driven initiatives can complement ANC efforts, addressing sociocultural factors, reducing stigma, and encouraging HIV-positive pregnant women to adhere to prevention measures.

### Global lessons for Uganda

Drawing insights from global experiences in the fight against MTCT of HIV can provide valuable lessons for Uganda. These lessons encompass strategies, innovations, and best practices that can inform and strengthen the country’s ongoing efforts to eliminate MTCT. Global initiatives emphasize the importance of early and universal HIV testing for pregnant women. Ensuring that all pregnant women in Uganda are tested early and universally for HIV is crucial. Early identification allows for timely interventions to prevent MTCT.^49^ Successful MTCT prevention programs globally integrate HIV services with routine maternal and child health services. Strengthening integration between HIV services and antenatal care in Uganda ensures a comprehensive approach, improving access and outcomes for pregnant women and infants.^50^ The Option B+ approach, providing lifelong ART to all pregnant and breastfeeding women living with HIV, has shown success in preventing MTCT. Continuing and expanding the implementation of Option B+ in Uganda ensures sustained ART coverage for HIV-positive pregnant women, optimizing prevention outcomes.^51^

Community engagement is a key component of successful MTCT prevention globally, reducing stigma and increasing awareness.^52^ Strengthening community engagement in Uganda fosters support, reduces stigma, and encourages participation in MTCT prevention initiatives. Global experiences highlight the importance of involving male partners in antenatal care and MTCT prevention.^53^ Promoting male involvement in Uganda contributes to a supportive environment, encouraging joint testing and enhancing family-focused MTCT prevention strategies. Successful MTCT prevention programs globally employ task shifting and invest in capacity building for healthcare workers.^54^ Implementing task-shifting strategies and continuous capacity building for healthcare workers in Uganda ensures a skilled workforce to deliver effective MTCT prevention services. The adoption of innovative technologies, such as mobile health apps and point-of-care diagnostics, has improved MTCT prevention globally.^55^ Leveraging innovative technologies in Uganda enhances accessibility, efficiency, and engagement in MTCT prevention programs, particularly in remote or resource-limited areas. Robust data management and surveillance systems globally contribute to the effective monitoring and evaluation of MTCT prevention programs.^56^ Strengthening data management and surveillance in Uganda ensures accurate tracking of progress, identifies challenges, and supports evidence-based decision-making. Global experiences emphasize the importance of cross-sectoral collaboration for comprehensive MTCT prevention. Strengthening collaboration between health, education, and community sectors in Uganda enhances the effectiveness of MTCT prevention strategies and addresses broader social determinants.^55^ Ensuring a continuum of care, from antenatal through postnatal periods, is vital for successful MTCT prevention globally. Establishing seamless transitions and follow-up care in Uganda ensures that interventions initiated during pregnancy are sustained, contributing to long-term prevention outcomes.^56^ Global successes in MTCT prevention underscore the importance of political will and sustained funding. Ensuring political commitment and sustainable funding in Uganda is crucial for the continued implementation and success of MTCT prevention programs.^55^

### Recommendations for Uganda

Ensure early and universal HIV testing for all pregnant women during their first ANC visit. Implement strategies to reach pregnant women who may not access traditional healthcare facilities, including those in remote or underserved areas. Further integrate HIV services with routine maternal and child health services, ensuring a holistic approach to healthcare for pregnant women. Facilitate collaboration between different healthcare providers to enhance the continuum of care for HIV-positive pregnant women. Continuously implement and promote the Option B+ approach, providing lifelong ART to all pregnant and breastfeeding women living with HIV. Address barriers to sustained ART adherence, including stigma, by implementing community-based interventions and educational campaigns. Implement programs to encourage male involvement in ANC and MTCT prevention initiatives. Provide education to men on the importance of joint testing, family planning, and supporting their partners throughout the pregnancy and postnatal period. Strengthen community engagement strategies to reduce stigma, increase awareness, and encourage active participation in MTCT prevention programs. Utilize community leaders and influencers to disseminate information and promote positive health-seeking behaviors.

Expand task-shifting initiatives and invest in ongoing capacity building for healthcare workers involved in ANC and MTCT prevention. Ensure that healthcare workers have the necessary skills and knowledge to provide comprehensive services and stay updated on evolving best practices. Explore and implement innovative technologies, such as mobile health apps and telemedicine, to enhance ANC services and engagement. Ensure that health workers are trained to effectively use and integrate technology into their daily practices. Improve data management and surveillance systems to track and evaluate the effectiveness of MTCT prevention programs. Implement regular monitoring and evaluation activities to identify areas for improvement and inform evidence-based decision-making. Facilitate collaboration between health, education, and community sectors to address social determinants of health and create a supportive environment for pregnant women. Engage multiple stakeholders, including policymakers, community leaders, and non-governmental organizations, in joint efforts to eliminate MTCT. Advocate for sustained political will at all levels of government to prioritize and support MTCT prevention initiatives. Work towards securing sustainable funding from domestic and international sources to ensure the continuous implementation and scaling up of successful programs. Strengthen the continuum of care by ensuring smooth transitions between antenatal, intrapartum, and postnatal periods. Implement strategies to improve follow-up care, including routine postnatal visits, to support ongoing health and well-being for both mothers and infants. Improve healthcare infrastructure, particularly in rural and underserved areas, to enhance access to ANC services and laboratory facilities. Invest in training and equipping healthcare facilities with the necessary resources for MTCT prevention, including diagnostic tools, medications, and skilled personnel.

### Future directions

Invest in ongoing research and development of innovative technologies for HIV testing, monitoring, and intervention in the context of MTCT prevention. Explore the potential of emerging technologies, such as artificial intelligence, advanced diagnostics, and digital health solutions, to further enhance the efficiency and effectiveness of MTCT programs. Embrace precision medicine approaches to tailor interventions based on individual patient characteristics, including viral genetics, to optimize treatment outcomes and prevent drug resistance. Explore the potential of personalized healthcare plans for pregnant women living with HIV, taking into account their unique medical history and circumstances. Expand the focus beyond HIV to address broader women’s health issues during pregnancy, including maternal nutrition, mental health, and obstetric care. Implement integrated healthcare models that consider the overall well-being of women during the perinatal period, contributing to healthier pregnancies and improved MTCT prevention outcomes.

Strengthen efforts to address social determinants of health, such as poverty, education, and gender inequality, which impact access to healthcare and contribute to the risk of MTCT. Collaborate with cross-sectoral partners to implement interventions that address the root causes of health disparities and vulnerabilities. Expand community-based interventions that empower local communities to actively participate in MTCT prevention efforts. Foster community-led initiatives that focus on awareness, education, and support, reducing stigma and creating an enabling environment for pregnant women living with HIV. Integrate sexual and reproductive health services seamlessly with MTCT prevention programs to address comprehensive healthcare needs. Ensure that family planning services, antenatal care, and other reproductive health components are embedded within MTCT prevention initiatives, promoting holistic maternal and child health. Develop targeted strategies to address the unique needs of young people, including adolescents and young adults, to prevent new HIV infections and ensure effective MTCT prevention. Implement youth-friendly services, education programs, and awareness campaigns to promote safe sexual practices and reduce the risk of transmission.

Strengthen surveillance systems and invest in data analytics to continuously monitor and evaluate the impact of MTCT prevention programs. Utilize real-time data to identify trends, target interventions, and make informed decisions for ongoing improvement. Continue to invest in capacity building and training programs for healthcare workers, ensuring a skilled and motivated workforce. Foster continuous professional development, especially in areas related to emerging technologies, evolving treatment protocols, and best practices in MTCT prevention. Encourage and support community-led research initiatives and implementation science projects to address local challenges and opportunities. Engage communities in the research process, ensuring that interventions are culturally sensitive, acceptable, and sustainable. Strengthen collaboration with global partners, sharing experiences and lessons learned in MTCT prevention. Participate in international forums and networks to stay updated on global advancements and contribute to the collective knowledge base. Anticipate and adapt to emerging challenges, including public health crises, climate change impacts, and potential disease outbreaks. Develop flexible and responsive strategies to ensure the resilience of MTCT prevention programs in the face of evolving circumstances.^57–62^

## Conclusion

In the pursuit of a MTCT-free Uganda, significant strides have been made, yet challenges persist. The collaborative efforts of government bodies, healthcare providers, communities, and international partners have laid a foundation for progress. Uganda has witnessed notable achievements in the prevention of MTCT. The implementation of Option B+, early testing initiatives, and integration of services within antenatal care have contributed to improved outcomes for pregnant women and their infants. Innovations such as point-of-care diagnostics and community engagement strategies have enhanced accessibility and reduced barriers. Central to the success of MTCT prevention is the empowerment of women and communities. Beyond addressing the biomedical aspects, there is a need for comprehensive strategies that consider the broader context of women’s health, social determinants, and community involvement. Ensuring the well-being of pregnant women living with HIV requires a multifaceted approach that goes beyond clinical interventions.

Sustainability is key to achieving long-term success. This involves building resilient healthcare systems, investing in capacity building, and adapting strategies to emerging challenges. The commitment to political will, continuous research, and learning from both successes and setbacks will be instrumental in sustaining progress. The fight against MTCT is not confined to national borders. Global collaboration, knowledge sharing, and collective efforts are essential. Uganda can contribute valuable insights to the global community while benefiting from experiences and innovations from around the world. Shared learning and collaboration amplify the impact of MTCT prevention initiatives. The vision for an MTCT-free Uganda requires unwavering commitment, innovation, and a holistic understanding of health. By addressing challenges, leveraging innovative technologies, and prioritizing the well-being of women and communities, Uganda can continue to advance towards the goal of eliminating MTCT. The journey is ongoing, and each step taken brings us closer to a future where every child is born free from the burden of HIV.

## Ethical approval

Not applicable.

## Consent

Not applicable.

## Source of funding

No funding was received for writing this review paper

## Author contribution

E.I.O. performed the following roles: conceptualisation, methodology, supervision, draft witing, editing and approval before submission.

## Conflicts of interest disclosure

The authors declare no conflict of interest.

## Research registration unique identifying number (UIN)

Not applicable.

## Guarantor

Not applicable.

## Data availability statement

Not applicable.

## Provenance and peer review

Not applicable.
